# Expression of human CD46 and trans-complementation by murine adenovirus 1 fails to allow productive infection by a group B oncolytic adenovirus in murine cancer cells

**DOI:** 10.1186/s40425-018-0350-x

**Published:** 2018-06-13

**Authors:** Janet Lei, Egon J. Jacobus, William K. Taverner, Kerry D. Fisher, Silvio Hemmi, Katy West, Lorna Slater, Fred Lilley, Alice Brown, Brian Champion, Margaret R. Duffy, Len W. Seymour

**Affiliations:** 1 Department of Oncology, University of Oxford, Oxford, OX3 7DQ UK; 2 Institute of Molecular Life Sciences, University of Zurich, Zurich, Switzerland; 3 PsiOxus Therapeutics Ltd, PsiOxus House, 4-10 The Quadrant, Barton Lane, Abingdon, Oxfordshire OX14 3YS UK

**Keywords:** Group B adenovirus, Mouse model, Adenovirus replication, Oncolytic viruses

## Abstract

**Background:**

Oncolytic viruses are currently experiencing accelerated development in several laboratories worldwide, with some forty-seven clinical trials currently recruiting. Many oncolytic viruses combine targeted cytotoxicity to cancer cells with a proinflammatory cell lysis. Due to their additional potential to express immunomodulatory transgenes, they are also often known as oncolytic viral vaccines. However, several types of oncolytic viruses are human-specific and the lack of suitable immune-competent animal models complicates biologically relevant evaluation of their vaccine potential. This is a particular challenge for group B adenoviruses, which fail to infect even those immunocompetent animal model systems identified as semi-permissive for type 5 adenovirus. Here, we aim to develop a murine cell line capable of supporting replication of a group B oncolytic adenovirus, enadenotucirev (EnAd), for incorporation into a syngeneic immunocompetent animal model to explore the oncolytic vaccine potential of group B oncolytic viruses.

**Methods:**

Transgenic murine cell lines were infected with EnAd expressing GFP transgene under replication-independent or -dependent promoters. Virus mRNA expression, genome replication, and late protein expression were determined by qRT-PCR, qPCR, and immunoblotting, respectively. We also use Balb/c immune-competent mice to determine the tumourogenicity and infectivity of transgenic murine cell lines.

**Results:**

Our results show that a broad range of human carcinoma cells will support EnAd replication, but not murine carcinoma cells. Murine cells can be readily modified to express surface human CD46, one of the receptors for group B adenoviruses, allowing receptor-mediated uptake of EnAd particles into the murine cells and expression of CMV promoter-driven transgenes. Although the early E1A mRNA was expressed in murine cells at levels similar to human cells, adenovirus E2B and Fibre mRNA expression levels were hampered and few virus genomes were produced. Unlike previous reports on group C adenoviruses, trans-complementation of group B adenoviruses by co-infection with mouse adenovirus 1 did not rescue replication. A panel of group B adenoviruses expressing individual mouse adenovirus 1 genes were also unable to rescue EnAd replication.

**Conclusion:**

Together, these results indicate that there may be major differences in the early stages of replication of group C and B adenoviruses in murine cells, and that the block to the life cycle of B adenoviruses in murine cells occurs in the early stage of virus replication, perhaps reflecting poor activity of Ad11p E1A in murine cells.

**Electronic supplementary material:**

The online version of this article (10.1186/s40425-018-0350-x) contains supplementary material, which is available to authorized users.

## Background

Oncolytic viruses are an emerging class of therapeutic agents with potent anti-cancer activity [1]. They self-amplify within infected cancer cells, releasing progeny virus particles upon cell death that can then infect neighbouring cancer cells. This not only causes direct tumour killing but, in some cases, can also cause exposure of pathogen-associated molecular patterns and danger-associated molecular patterns that can activate the host immune system [2–4]. Oncolytic viruses are also often engineered to express immune-stimulatory transgenes within tumour cells [5], earning them the epithet ‘oncolytic vaccines’.

Adenoviruses represent a particularly promising class of oncolytic viral vaccines. However, despite the wealth of research into adenovirus cell and molecular biology, one of the major bottlenecks in oncolytic adenovirus research is the lack of suitable immune-competent animal tumour models to study the immunobiology, replication, and oncolytic vaccine activity of different groups of adenoviruses in vivo [6, 7]. Importantly, the presence of a functional adaptive immune system is essential to evaluate the effects of immune stimulation on anticancer activity. An ideal animal tumour model would be syngeneic, avoiding the use of xenografted human tissues and obviating the need for immunocompromised animal hosts.

One approach could be to make use of murine adenoviruses (MAVs) as relevant surrogates for human agents, allowing their study in syngeneic murine models. However the most extensively-studied MAV, MAV-1, causes fatal disease in C57BL/6 immune-competent mice, making it a poor proxy for the much milder effects of adenovirus infection observed in humans [8].

An alternative approach is to identify animal cells that are permissive to human adenoviruses. Murine models are the most widely used for cancer research; however human adenoviruses typically show little activity in murine cells [9, 10]. Although some murine tumour cell lines are semi-permissive for type 5 adenovirus (Ad5), full replication is generally limited to a select number of cell lines [6, 7, 9]. Group B adenoviruses (such as Ad11p) show no infection at all, likely because the main receptor for group B adenoviruses (CD46) is not expressed in most murine or hamster cells [11]. Syrian hamsters have been reported to support Ad5 replication [12], but the range of cancer models available for work in hamster models is very small compared to those available for mice. Cotton rats are used in preclinical testing of Ad5, but the same issues of availability of cancer models also apply here [13].

The inability of human adenoviruses to replicate in murine cells reflects at least two fundamental problems. First, murine cells have variable expression levels of the receptors required for human adenovirus entry into the cell [14]. Whereas group C adenoviruses use predominantly the coxsackie and adenovirus receptor (CAR), group B adenoviruses use either CD46 or desmoglein 2 and sometimes both [15–17].

Second, although Ad5 is able to gain entry into murine cells and successfully complete the early part of its replication cycle (including genome replication and mRNA production), translation of late viral mRNA into protein is reported to be inefficient [18]. Unlike translation of cellular mRNA, which usually occurs via 5′ ribosome scanning of capped mRNA for the start codon, translation of late adenoviral mRNA normally occurs via ribosome shunting, an alternative cap-dependent process involving ribosome jumping to downstream start codons [19–21]. In the case of Ad5, the defect can be complemented, or rescued by expression of another protein, by co-infecting cells with MAV-1, suggesting that some MAV-1 components allow translation of Ad5 late mRNA [18]. The defect can also be partially complemented by overexpression of the Ad5 L4-100 K protein, which plays a key role in hijacking the translation machinery to drive ribosome shunting and translation of late proteins. This partial rescue could be a result of a sub-optimal interaction between human adenovirus L4100 K and the murine translation machinery, raising a clear possibility that MAV-1 L4-100 K will rescue Ad5 replication in murine cells even more efficiently.

Enadenotucirev (EnAd) is an oncolytic adenovirus formed as a chimera of the two group B adenoviruses Ad3 and Ad11 [22]. A product of bioselection in HT-29 colorectal cancer cells, EnAd shows impressive selectivity for replication in human carcinoma cells, including in a co-culture of cancer and normal cells in vitro [23]*,* and has shown a promising targeting and safety profile in an early clinical trial [24]. EnAd has recently been shown to be an efficient vector for cancer-selective expression of immune-targeting biologics [25] and can be delivered from the bloodstream into the tumour following systemic administration to humans [24, 26, 27]. Although xenografted human tumours can be used to assess direct oncolytic cytotoxicity in mice, the lack of a syngeneic (immune-competent) model limits preclinical assessment of potential cancer vaccine activity. Though a panel of assays in appropriate cell lines, immune-deficient mice, and patient biopsies could be used as an alternative to immune-competent mice [23], establishment of such a panel for each new candidate virus could prove to be time-consuming and challenging.

Here, we describe a series of studies aiming to modify murine cells to support productive group B adenovirus infection, using EnAd as a model virus. We first assess EnAd replication in a panel of human carcinoma cells and then show that a panel of murine cells can be modified to express human CD46, enabling entry of virus particles into the cell and expression of GFP transgene encoded within the EnAd genome under control of the CMV immediate-early promoter. However, there was neither virus replication-linked reporter gene expression (using the adenovirus major late promoter) nor any sign of oncolysis. While E1A mRNA was expressed at similar or even higher levels in CD46-expressing murine cells compared to A549 human lung carcinoma cells, expression levels of E2B and Fiber mRNA were markedly lower in murine compared to human cells. Co-infection with MAV-1 did not affect EnAd genome replication nor restore adenovirus major late promoter (MLP)-driven GFP expression. Finally infection of CD46-expressing murine cells with a panel of recombinant EnAd expressing each of the MAV-1 open reading frames also did not enhance MLP-driven GFP expression, though replication-independent GFP expression was improved in some cases. Our study shows that, while the addition of human CD46 to murine cells alleviates one barrier to group B adenovirus replication, other factors within the cell that inhibit group B adenovirus replication remain to be defined.

## Methods

### Mammalian cell culture

Human colorectal carcinoma cells (DLD-1, HT29, HCT-116, SW480, SW620), human lung carcinoma (A549), human prostate carcinoma (PC-3, DU145, LNCaP), human prostate carcinoma (Panc-1, Capan-2, BxPC3, CFPAC-1), human breast carcinoma (MDA-MB-231, MDA-MB-453, BT-20. MCF-7) human ovarian carcinoma cells (SKOV3, OVCAR3, PA-1, Caov3), human bladder carcinoma (RT4, T24, HT-1376, UM-UC-3), human embryonic kidney cells (293, 293 T), murine colorectal carcinoma (CT26, CT26-CD46, CMT93, CMT93-CD46) murine lung carcinoma (CMT64, and CMT64-CD46) and murine breast cell (NMuMG, NMuMG-CD46) were cultured in either RPMI or DMEM supplemented with 10% fetal calf serum and 1% penicillin-streptomycin (herein known as normal culture medium) at 37 °C and 5% CO_2_. All cell lines were obtained from ATCC, apart from PA-1, SKOV3, and SW480 which were obtained from ECACC.

### Lentivirus transduction

Murine NMuMG, CMT93, and CMT64 cells stably and constitutively expressing human CD46 were engineered using a human CD46-encoding lentivirus vector, as described previously [23]. Cleared supernatant was supplemented with 8 μg/ml polybrene and added to murine cells seeded in a 10 cm dish. Fresh culture medium was added at 24 h post infection (p.i.). At 3 days post infection, the medium was changed to selection medium containing 2 μg/mL puromycin. Single colonies were isolated by limiting dilution in selection medium and tested for CD46 expression by flow cytometry using a PE-conjugated αCD46 antibody (1:100, clone: TRA-2-10, BioLegend) or the corresponding PE-conjugated IgG1κ isotype control. Clonally derived recombinant cell populations were used for the rest of the study.

### Modification of EnAd to encode transgenes

The genome of EnAd was modified using the parental vector ColoAd2.4 [28]. Transgenes were amplified by PCR using MAV1 genomic DNA extracted from MAV1-infected CMT93 cells as a template. Primers for transgene amplification were designed using the complete genome sequence of MAV1 (AC_000012.1) to amplify the annotated protein-coding regions from each gene, including protein-coding regions generated through alternative splicing. The reverse primer for every protein-coding region was designed to encode the octapeptide DYKDDDDK (FLAG-tag) for detection. Primer sequences are given in Additional file 1. Amplicons were gel-extracted using a Gel Extraction Mini Kit (QIAGEN) and cloned into the multiple cloning site in the shuttle vector pSF-CMV (Oxford Genetics) before transforming into *E. coli* DH10β chemically competent cells (New England Biolabs). Correct transgene insertion was confirmed by restriction digest and Sanger sequencing (GATC Biotech). Using ColoAd_F and ColoAd_R, the transgene was transferred into ColoAd2.4 by Gibson assembly using 2× HiFi Master Mix (New England Biolabs).

Recombinant EnAd was rescued by digesting plasmids containing the entire EnAd genome using AscI. Linearised fragments were precipitated using 0.6 volumes isopropanol and centrifuging for 30 min at 4 °C. Fragments were resuspended in ddH_2_O and 5 μg DNA was transfected into 1 × 10^6^ HEK293A cells in a T25 flask using Lipofectamine 2000. Cells were left until plaques were visible. Supernatant was collected and viruses were plaque purified and tested for transgene expression using a FLAG-tagged antibody in an immunoblot. Viruses were selected for purification by cesium chloride banding, as described in [29].

### Infection studies

Infection studies were performed in normal culture medium for 2 hours at 37 °C before changing the medium for fresh normal culture medium. Cells were incubated at 37 °C for the indicated number of days before harvesting and analysis. GFP-expressing cells were visualised by brightfield and fluorescence microscopy using a Zeiss Axiovert 25 and an ebq 100 isolated mercury lamp power source.

### Flow cytometry

Cells were analysed by flow cytometry for expression of CD46 or a GFP transgene. Cells were harvested by trypsinisation and transferred to a 96-well V-bottom plate. For GFP analysis, cells were pelleted by spinning at 400 x g for 5 min before resuspending in 4% paraformaldehyde. Cells were incubated for 10 min at room temperature before washing with staining buffer (0.5% bovine serum albumin and 2 mM EDTA in PBS). For CD46 analyses, cells were pelleted and resuspended in staining buffer containing 0.5 μg/100 μl PE anti-CD46 (BioLegend 352,402) or the corresponding PE Mouse IgG1, κ isotype control (BioLegend 400,114) and incubated at room temperature for 30 min in the dark. Cells were then washed once with MACS buffer and resuspended in staining buffer for measurement on an Attune NxT Flow Cytometer (Thermo Fisher Scientific). Data was analysed using FlowJo V.10.

### qPCR

EnAd genomes were measured by quantitative PCR using primers and probes specific for the hexon or E3 gene. Genomic DNA was extracted from harvested cells using the PureLink Genomic DNA Purification Kit (Life Technologies). EnAd genomes per 30 ng DNA were quantified in a 20 μl qPCR reaction consisting of 2× qPCRBIO Probe Mix Hi-Rox (PCR Biosystems), and 10 μM each of forward primer (5′- TACATGCACATCGCCGGA-3′), reverse primer (5’-CGGGCGAACTGCACC-3′), and hexon probe ([6FAM]-CCGGACTCAGGTACTCCGAAGCATCCT-[TAM]. E3 was detected using a forward primer (5’- ATCCATGTCTAGACTTCGACCCAG -3’), reverse primer (5’- TGCTGGGTGATAACTATGGGGT -3’), and E3 probe ([6FAM]- ATCTGTGGAGTTCATCGCCTCTCTTACG-[TAM]). Cycling conditions were as follows: one cycle at 95 °C for 2 min, followed by 40 cycles at 95 °C for 5 s and 60 °C for 30 s. C_T_ values from known quantities of virus particles were used to calculate a standard curve.

### Reverse transcriptase-PCR

Cells infected with EnAd encoding MAV1 ORF transgenes were tested for mRNA expression by RT-PCR. Total RNA was extracted using the RNeasy Mini Kit (QIAGEN) with on-column DNA digestion. cDNA was generated using the QuantiTect Reverse Transcription Kit (QIAGEN). Coding regions were amplified using primers binding to the 5’-UTR (post_CMV_seq_F, 5’-CCATCCACTCGACACACCC-3′) and 3’-UTR (pre_polyA_seq_R, 5′- GTGAGCTGAAGGTACGCTG-3′). Amplicons were separated on a 1% agarose-TAE gel by electrophoresis.

### Reverse transcriptase-quantitative PCR

EnAd E1A, E2B, and Fibre mRNA expression were measured by RT-qPCR. Total RNA was extracted using the RNeasy Mini Kit (QIAGEN) with on-column DNA digestion. cDNA was generated using the QuantiTect Reverse Transcription Kit (QIAGEN). mRNA copies per 30 ng cDNA were quantified in a 20 μl qPCR reaction consisting of 2× qPCRBIO Probe Mix Hi-Rox (PCR Biosystems), and 10 μM each of forward primer, reverse primer, and probe (Table 1). Cycling conditions were as follows: one cycle at 95 °C for 2 min, followed by 40 cycles at 95 °C for 5 s and 60 °C for 30 s. C_T_ values from known copy numbers of each gene were used to calculate a standard curve.

**Table 1:** Primers and probes used for RT-qPCR. Sequences are given as 5′-3′. Probes are tagged with 6-FAM at the 5′-end and BHQ1 at the 3′-end

Gene	Forward primer	Reverse primer	Probe
E1A	CCATCTCCTGATTCTACTACC	CCGTGTACTCAAGTCCAA	TAAGCCTGGGAAACGTCCAGCAGT
E2B	CTCTTCAATGATGTTACTTTCG	GTAGCGAAGCGTGAGTAAG	AGGCTCCCTGTTCCCAGAGTTGGA
Fibre	ACCGAAGAGCAATAAATG	TCGTCTTCTCTGATGTAG	TCGTATAACTTGGTCCTGGAACACA

### Immunoblotting

Protein expression in infected cells was analysed by immunoblotting. Infected cells were harvested by removing the supernatant from cell cultures and rinsing gently with PBS. Cells were lysed by adding Pierce RIPA buffer supplemented with 1 x protease inhibitor directly to the cell monolayer and incubating at room temperature for 5 min. Lysates were scraped and transferred into 1.5 mL Eppendorf tubes and incubated with 2.5 U Benzonase for 30 min at room temperature. Lysate concentrations were measured by the QuantiPro BCA Assay Kit (Sigma-Aldrich). Samples containing 40 μg of each protein lysate in 1× Laemmli sample buffer were heated at 95 °C for 5 min. Proteins were separated on a 4-20% Mini-PROTEAN TGX Precast Protein Gel and transferred onto a 0.2 um nitrocellulose membrane using the wet blot method. Late group B adenovirus structural proteins were visualised by a polyclonal goat antibody against adenovirus (ab3685, Abcam) and a mouse monoclonal anti-goat IgG conjugated to horseradish peroxidase (sc-2354, Santa Cruz Biotechnology). FLAG-tagged proteins were visualised using Direct-Blot™ HRP anti-DYKDDDDK Tag Antibody (BioLegend). Membranes were incubated with SuperSignal West Dura Extended Duration Substrate (Thermo Fisher) before exposing on Amersham Hyperfilm ECL (GE Healthcare Life Sciences).

### Animal experiments

Animal experiments were carried out in accordance with the UK Home Office guidelines under the Animals (Scientific Procedures) Act 1986. CT26 (1 × 10^6^ cells, *n* = 3), CT26-CD46 (1 × 10^6^ cells, *n* = 5; 5 × 10^6^, n = 5) were inoculated subcutaneously on right flank of Balb/c mice. HCT116 (2 × 10^6^ cells, n = 3) were inoculated subcutaneously on right flank of nude mice. Once a palpable tumour was apparent, tumour growth was monitored until a volume of 70-150 mm^3^ (V = LxWxDxpi;/6) was reached. All animals bearing palpable tumours were treated with a multi-centre intratumoural injection of 5 × 10^9^ particles of EnAd-CMV-Luc virus in 50 μL PBS. Administration of virus was performed in a procedure chamber equipped with a HEPA filter. IVIS imaging was performed before and after treatment (4-6 imaging sessions) to monitor virus-mediated expression of firefly luciferase. Before imaging, 150 mg/kg D-luciferin dissolved in 100 μL sterile PBS was administered subcutaneously. Animals were anesthetised for the imaging procedure with isoflurane. Tumours were harvested after confirmation of virus-mediated luciferase expression. Mice were sacrificed when tumours exceeded a volume of 1000 mm^3^ or, for tumours that did not exceed this volume, at 31 days post-transplantation. Tumours were then excised and fixed in paraffin for immunohistochemistry.

### Immunohistochemistry

Tissues were fixed in 10% normal buffer saline prior paraffin embedding. Four-micron tissue slices were deparaffinised and rehydrated. Epitope retrieval was performed at 60 °C for 20 min using Epitope Retrieval 2 (AR9640, Leica). Tissues were then stained with rabbit anti-CD46 at 0.078 μg/mL for 60 min (1:1000, ab108307, Abcam). Detection of primary antibody was performed using the Polymer Refine Detection Horseradish peroxidase for 8 min (DS9800, Leica). Between antibody or polymer incubation steps, tissues were washed twice with Wash buffer (AR9590, Leica). Tissues were counterstained with haematoxylin. Slides were processed using the Bond-Max (Leica).

## Results

### EnAd show high levels of replication in human carcinoma cells, but does not replicate in murine carcinoma cells

**Fig. 1:**
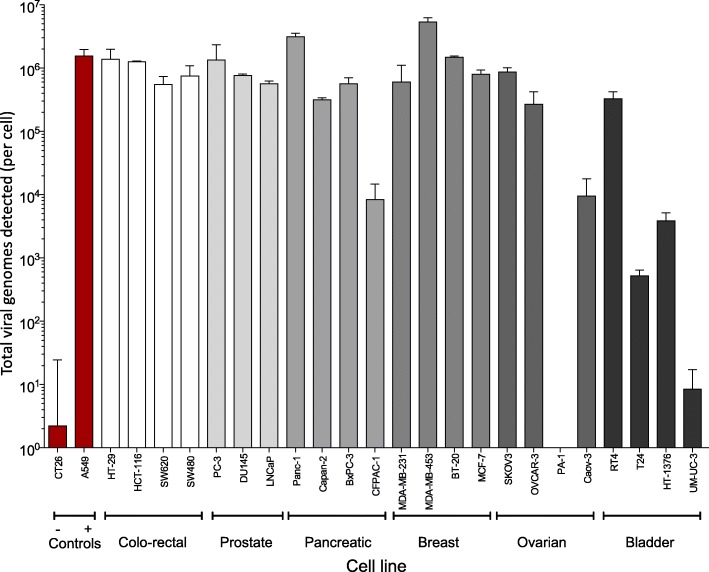
EnAd genome replication in a multi-indication human cell line panel. Duplicate cultures of 23 test tumour cell lines, plus positive (A549, small cell lung carcinoma) and negative (CT26, mouse colon carcinoma) control cell lines were inoculated with 1ppc of EnAd or assay media alone (uninfected control) and cultured at 37 °C, 5% CO_2_ for 3, 4, 8 or 11 days. At each time point, supernatants and cell lysates were harvested and frozen, before DNA extraction. qPCR was then run (triplicates) using the E3 primer/probe set. Data was background subtracted: mean genome quantity for each uninfected control triplicate was subtracted from the corresponding individual EnAd values. Genome quantity per cell was then calculated, and the mean of the two EnAd qPCR triplicates determined. Lysate and supernatant results were then combined to give a total genome detection value. The mean of the duplicate values at the time point showing maximal expression was plotted in the graph, with the SD represented by error bars. For the positive control cell line (A549), mean and SD was calculated across all runs at day 4 (*n* = 10 qPCR triplicates). For the negative control cell line (CT26), mean and SD was calculated across all runs at day 11 (*n* = 9 qPCR triplicates). Maximal genome expression was at day 8 for the following cell lines: HCT-116, SW620, SW480, PC-3, CFPAC-1, Caov-3, HT-1376, and at day 11 for the following cell lines: HT-29, DU145, LNCaP, Panc-1, Capan-2, BxPC-3, MDA-MB-231, MDA-MB-453, BT-20, MCF-7, SKOV3, OVCAR-3, PA-1, RT4, T24, UM-UC-3. Viral genome was undetectable at any time point with PA-1 cells

A range of human carcinoma cells were compared for their permissivity to EnAd replication. These included human carcinoma cells from a variety of origins and also murine CT26 colorectal carcinoma. A549 cells were used as a positive control, as EnAd is known to infect them well and replicate efficiently. However many of the human carcinoma cell lines showed similar levels of virus infection and replication, some even higher than A549 (Fig. 1). Human cells that did not support such high levels of EnAd infection included PA-1 cells, which were subsequently found to originate from an ovarian teratocarcinoma and are therefore of stem cell origin, and UMUC-3 bladder carcinoma cells. CT26 murine colorectal carcinoma cells were included for comparison, and showed no appreciable virus genome replication at all. This suggests that either the virus does not enter these cells, or the murine cellular machinery is completely unsuitable to support EnAd replication.

### NMuMG cells can express surface CD46 at levels comparable to human cell lines

**Fig. 2:**
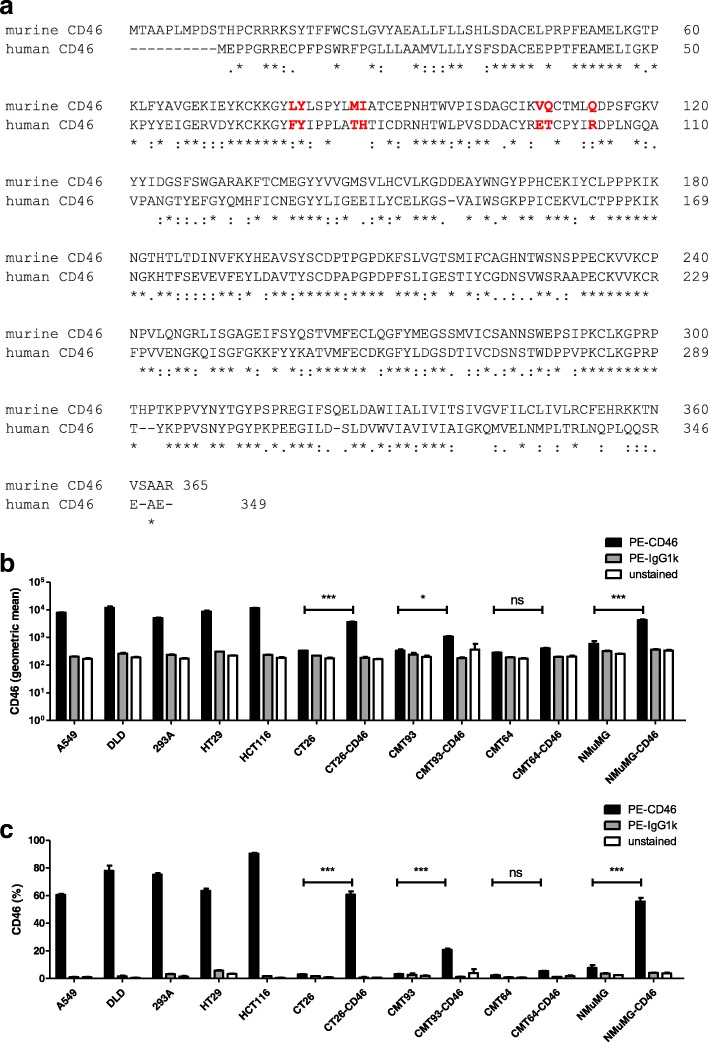
Human CD46 can be stably expressed on murine cells. **a.** Clustal Omega protein sequence alignment of murine and human CD46 (GenBank accession BAA31859.1 and BAA12224.1, respectively). The murine and human CD46 protein sequences have a similarity of 49%. Red bold font indicate binding sites of human CD46 and Ad11 fiber knob, as described by Persson et al. [31]. **b** and **c**. Murine cells were transduced with a lentiviral vector to generate cell lines stably expressing human CD46. CD46 surface expression in murine and human cell lines was compared by flow cytometry by staining 1 × 10^6^ cells/sample using PE-human CD46 or the corresponding isotype control. Data show (**b**) the geometric mean of the intensity of CD46 expression and (**c**) the proportion of cells expressing CD46. Data represent biological triplicates, shown as mean ± SEM. Significance between the parental and corresponding CD46 cell line was assessed using one-way ANOVA with Tukey’s Post Hoc analysis. *, *p* < 0.05; ***, *p* < 0.001; ns, not significant

Murine cell lines have previously been shown to have varying levels of permissiveness to infection by Ad5, a group C adenovirus. However, there have been no reports of infection of murine cells by group B adenoviruses. Unlike group C adenoviruses, group B adenoviruses utilise CD46 and desmoglein-2 as entry receptors [15, 16]. Whereas murine and human CAR are both widely expressed and have 91% sequence homology in their extracellular domain, making murine CAR suitable as a receptor for group C adenoviruses [14, 30], murine CD46 and human CD46 have major differences in the key residues found previously to be involved in binding of the adenovirus fibre knob during attachment (Fig. 2a, [31]). In addition murine CD46 is restricted predominantly to testicular cells, therefore, it is unlikely that murine CD46 could serve as a receptor for human adenoviruses in most cancer cell types. We therefore used lentivirus transduction to stably express human CD46 on several different murine cell lines. Human CD46 was cloned from DLD human colon adenocarcinoma cells into a self-inactivating lentiviral genome, which was rescued by cotransfection with packaging plasmids. The lentivirus was used to transduce the murine cell lines CMT64 (lung carcinoma), CMT93 (rectum polyploidy carcinoma), NMuMG (mammary gland), and CT26 (colon carcinoma). Transduced cell populations were selected by puromycin for lentivirus integration and serially titrated to obtain single clones. The levels of human CD46 expression on these recombinant cell lines was compared to a panel of human cell lines using flow cytometry. CD46 expression levels showed considerable variation between the human cell lines, with DLD-1 and HT-29 cells expressing the highest levels and A549 and 293A cells expressing moderate levels (Fig. 2b). As expected, unmodified CMT64, CMT93, NMuMG, and CT26 murine cells did not express any human CD46. However, the stably transduced cells showed expression of human CD46 on the cell surface, confirming that the protein is expressed and embedded into the plasma membrane. Amongst murine cells, NMuMG-CD46 expressed the highest levels of human CD46, while CT26-CD46 expressed moderate levels, CMT93-CD46 had only slight but still significant levels of human CD46 expression over the corresponding parental control cell lines. CMT64-CD46 cells did not express CD46 at levels above the parental cell line. The results are also reflected in the number of cells expressing human CD46 on the cell surface above the background level, as determined by untransduced murine cells (Fig. 2c). These results suggest that murine cells contain all the necessary machinery to express human CD46 on their surface.

### NMuMG cells stably expressing human CD46 can be infected by EnAd, a chimeric group B adenovirus

**Fig. 3:**
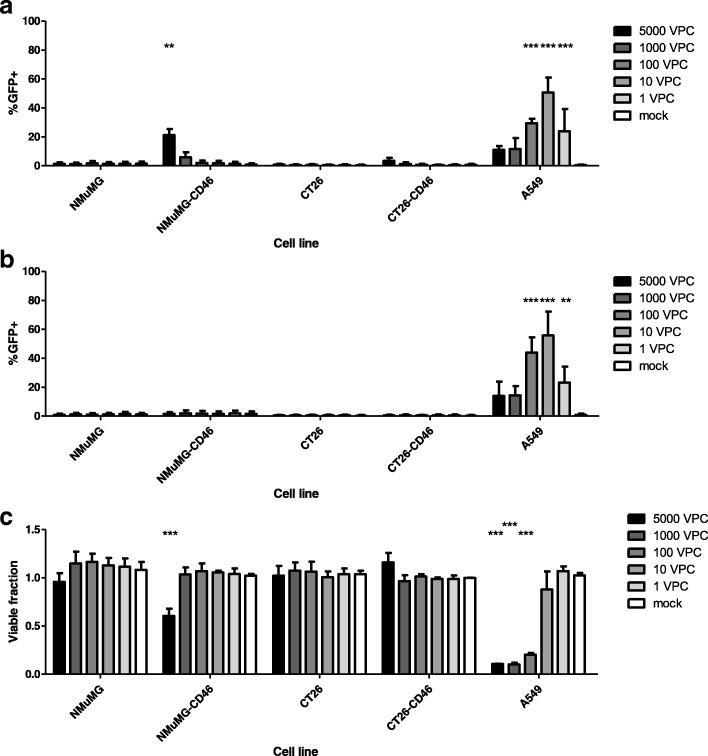
Expression of human CD46 allows EnAd to undergo replication-independent GFP but not adenovirus major late promoter-driven protein expression. NMuMG, NMuMG-CD46, CT26, CT26-CD46, and A549 cells were seeded at 1 × 10^4^ cells/well in 96-well plates before infection with 5000, 1000, 100, 10, or 1 virus particles/cell (VPC) of either (**a**) EnAd-CMV-GFP or (**b**) EnAd-SA-GFP, or mock-infected. Cells were harvested at 5 days post-infection and analysed by flow cytometry for GFP expression. **c** At 5 days post-infection, the surviving fraction of EnAd-CMV-GFP-infected cells compared to mock-infected was measured by MTS. Data represent means of three independent experiments, shown as mean ± SEM. Significance within each treatment was assessed using two-way ANOVA with Bonferroni correction compared to mock-infected cells. **, *p* < 0.01; ***, *p* < 0.001

To determine whether human CD46 expression enables infection of murine cells by group B adenovirus, we incubated NMuMG-CD46, CT26-CD46 and their corresponding parental cell lines with EnAd expressing GFP under the control of the CMV immediate-early promoter (EnAd-CMV-GFP) or under a splice acceptor (SA) site of the adenovirus MLP (EnAd-SA-GFP). In the former configuration, GFP expression controlled by the CMV immediate early promoter is expected to occur soon after the entry of the incoming virus into the nucleus, independent of whether the virus can complete its full replicative life cycle. In contrast, with EnAd-SA-GFP, GFP expression should occur only during the late phase of virus infection, and is therefore coupled to the virus replication cycle. Five days post-infection, the fraction of GFP-positive cells was measured by flow cytometry. No appreciable virus transgene expression was observed with either virus in any parental murine cell type. However both NMuMG-CD46 and CT26-CD46 cells showed measurable levels of GFP expression using EnAd-CMV-GFP, indicating the virus had successfully entered the cell and reached as far as the nucleus (Fig. 3a). In NMuMG-CD46 cells, the level of EnAd-CMV-GFP expression was approximately one third that seen in A549 cells, although it was lower in CT26-CD46 cells. These levels of expression are likely to reflect several factors, including the lower levels of CD46 expression achieved in murine cells compared to A549 (Fig. 2c).

In contrast, the expression of EnAd-SA-GFP expression was barely above background in either murine cell type (Fig. 3b), although in the human A549 cells levels of expression reached as high as with EnAd-CMV-GFP. At very high virus doses, rapid death of A549 cells led to lower-than-expected fractions of fluorescent cells due to the abundance of debris and dead cells. This result suggests that some virions are able to translocate to the nucleus in both murine cell types, leading to some transcriptional activity of the CMV promoter, but that MLP-driven gene expression of this virus is severely impaired even in these human CD46-expressing murine cells compared to A549 cells.

To determine whether CD46-expressing murine cells could be lysed by EnAd as part of its normal life cycle, cytotoxicity was assessed using an MTS assay. Cytotoxic activity was not observed with any of the murine cell lines, regardless of human CD46 expression (Fig. 3c). Together with our results with EnAd-CMV-GFP and EnAd-SA-GFP, we conclude that, though CD46 enables uptake of EnAd into murine cells, there are other factors at play that restrict the permissivity of murine cells to human adenovirus infection.

### Virus genome replication and late protein expression are inhibited in mouse cells

**Fig. 4:**
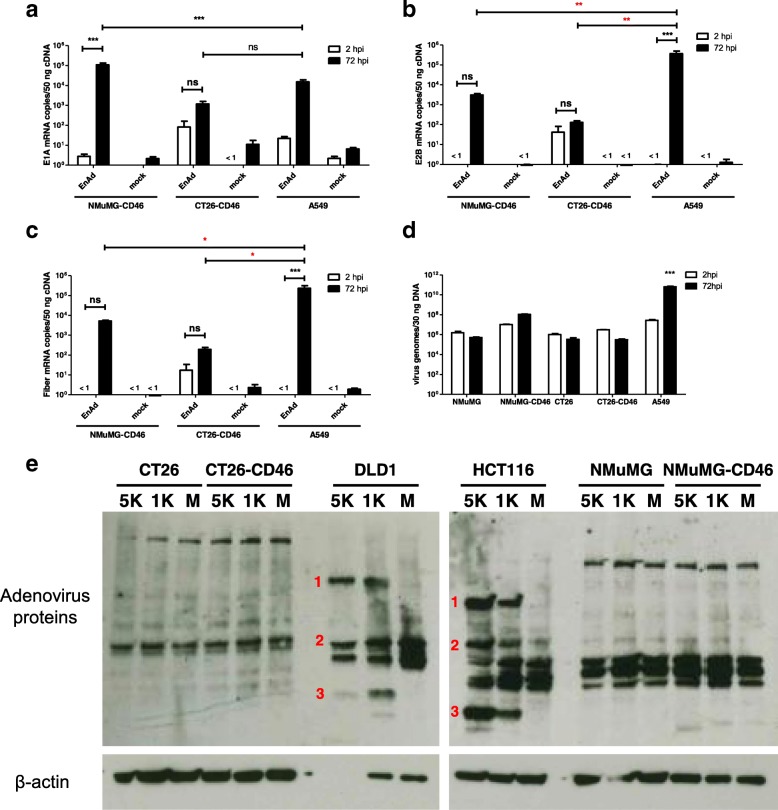
The blockade to EnAd replication in murine cells is multifactorial. **a** NMuMG-CD46, CT26-CD46, and A549 cells were seeded at 1 × 10^5^ cells/well in a 24-well plate before infection with 5000 EnAd-CMV-GFP particles/cell or mock-infected. At 2 and 72 h post-infection, cells were washed and harvested for RNA extraction and subsequent cDNA conversion. Copy numbers of Ad11 (**a**) E1A, (**b**) E2B, and (**c**) Fiber mRNA in 50 ng cDNA were quantified by qPCR using Taqman probes. A synthetic oligonucleotide specific to the PCR product was used as a standard. “< 1” indicates conditions with values below 1 copy/50 ng DNA. **d** NMuMG, NMuMG-CD46, CT26, CT26-CD46, and A549 cells were seeded at 2 × 10^5^ cells/well in a 12-well plate before infection with 5000 EnAd-CMV-GFP particles/cell or mock-infected. At 2 and 72 h post-infection, cells were wash and harvested for genomic DNA extraction. EnAd genome copies in 30 ng of genomic DNA were quantified by qPCR using Taqman probes. **e** NMuMG, NMuMG-CD46, CT26, CT26-CD46, HCT116, and DLD1 cells were seeded at 1 × 10^6^ cells/well in a 6-well plate before infection with 5000 (5 K) or 1000 (1 K) EnAd-CMV-GFP particles/cell or mock-infected (M). At 72 h post-infection, cells were lysed and analysed for protein expression by immunoblotting. Late group B adenovirus structural proteins were visualised by a goat anti-adenovirus polyclonal antibody. Red text on the immunoblot are labels for 1, adenovirus hexon (105.3 kDa); 2, penton (62.3 kDa); and 3, fiber (36.1 kDa). Data represent biological triplicates, shown as mean ± SEM. Significance within each treatment was assessed using two-way ANOVA with Bonferroni’s Post Hoc analysis compared to infected A549 cells at 72 h post-infection (A-C) or to each corresponding measurement at 2 h post-infection (D). **, *p* < 0.01; ***, p < 0.001; ns, not significant. Black asterisks represent a significantly higher levels compared to infected A549 cells at 72 h post-infection; red, significantly lower levels

Previous studies on Ad5 replication in mouse cells showed that late protein expression, but not early or late mRNA expression, is repressed [18]. To test whether expression of EnAd mRNA is repressed in mouse cells, mRNA was extracted from EnAd-treated NMuMG-CD46 and CT26-CD46 cells 2 and 72 h post-infection, and the number of copies of Ad11 E1A, E2B, and Fibre mRNA were measured (Fig. 4a-c). Surprisingly the copy numbers of E1A mRNA at 72 h were higher in NMuMG-CD46 than A549 cells (Fig. 4a). In contrast E2B mRNA levels were lower in NMuMG-CD46 cells than in A549, raising the possibility there is a block in the virus life cycle that occurs between transcription of E1A and E2B (Fig. 4b). E2B encodes adenovirus polymerase and terminal protein, hence genome replication is likely to be severely impaired if E2B expression is inhibited. It was not surprising, therefore, that levels of Ad11 Fiber mRNA were also reduced in mouse cells, suggesting that inhibition of early mRNA expression had knock-on effects on late mRNA expression, as previous studies found with Ad5 (Fig. 4c).

To confirm our expectation that the low levels of E2B mRNA would impact on viral genome replication, murine and human cells were incubated with EnAd-CMV-GFP or mock-infected. Cells and supernatants were harvested for genomic DNA extraction after 2 and 72 h p.i. and virus genome copies were measured by qPCR using Ad11 hexon-specific primers and Taqman probe. Genome copies increased only slightly in NMuMG-CD46 cells and not at all in CT26-CD46 cells between 2 and 72 h p.i. (Fig. 4d).

To determine whether the decrease in early and late viral mRNA levels translates into decreased late protein expression, murine and human cells were incubated with 5000 or 100 EnAd-CMV-GFP particles/cell or mock-infected. Cells were lysed 5 days p.i. and separated by SDS-PAGE. Blots were probed using a polyclonal antibody against adenovirus structural proteins. In murine cells no adenovirus structural proteins were expressed at detectable levels, independent of CD46 expression (Fig. 4e). In contrast, high levels of adenovirus structural proteins were seen in A549 cells. Our results show that while E1A may be transcribed effectively in CD46-transduced murine cells, subsequent aspects of viral replication, from E2B transcription onwards, are substantially inhibited.

### Assessment of EnAd infection of CD46-expressing murine cells in syngeneic host mice

**Fig. 5:**
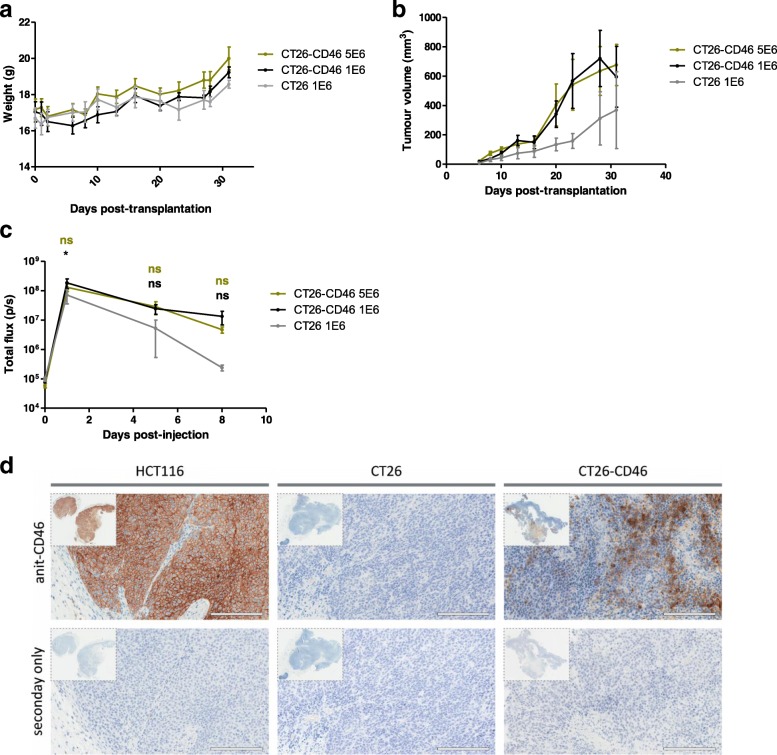
CT26-CD46 cells can establish tumours in immunocompetent mice. Balb/c mice were injected subcutaneously with either 1 × 10^6^ CT26 or CT26-CD46 cells or 5 × 10^6^ CT26-CD46 cells. Athymic mice inoculated with 2 × 10^6^ HCT116 cells using the same method were used as a positive control for luciferase expression. When tumours reached a volume of 70-150 mm^3^, 5 × 10^9^ VP of EnAd-CMV-Luc were injected intratumourally. Luciferase expression was monitored over 2 weeks following virus injection. **a** Mouse weights were monitored at regular intervals for 31 days. **b** Tumour volumes for each treatment group were measured once palpable tumours were apparent. **c** Mice were imaged for luciferase expression at the indicated time points after intratumoural injection with EnAd-CMV-Luc using an IVIS imager. Conditions were compared to CT26 tumours using one-way ANOVA. *, *p* < 0.05; ns, not significant. **d** Paraffin-embedded tumours were sliced into 4 μm slices and stained for human CD46 expression and counterstained with haematoxylin. Scale bars represent 200 μm

Balb/c mice were injected subcutaneously with either 1 × 10^5^ CT26 or CT26-CD46 cells or 5 × 10^5^ CT26-CD46 cells. We did not observe any weight loss (Fig. 5a) or loss of tumourigenicity in CT26 cells expressing human CD46 expression, which could arise from immunological rejection in immunocompetent mice (Fig. 5b). Indeed the CT26-CD46 tumours grew more quickly than the unmodified CT26, perhaps reflecting the clonal selection they had undergone. When tumours reached a volume of 70-150 mm^3^, 5 × 10^9^ VP of EnAd-CMV-Luc were injected intratumourally. The presence of CD46 on the cell surface gave only a small increase in luciferase expression in the short term, most likely indicating that infection following direct intra-tumoural injection of virus can be less receptor-dependent. The duration of luciferase expression appeared to be more sustained in CT26-CD46 tumours compared to unmodified CT26 tumours, and after 8 days there was a 50-fold differential (Fig. 5c). These results suggest that CT26-CD46 tumours may allow better virus entry into engrafted tumours and sustained transgene expression, although larger studies are required to confirm these results.

Immunohistochemistry of excised tumours showed that human CD46 staining is confined to the cellular membrane in both HCT116 and CT26-CD46 tumours. Expression of human CD46 in HCT116 tumours in a xenograft model was ubiquitous, except for mouse stromal and endothelial tissue. No CD46 expression was present in CT26 tumours. CD46 levels vary throughout CT26-CD46 allografts, suggesting expansion of cells with differing levels of CD46 during tumour implantation. Expression of human CD46 in this allograft 23 days post- tumour inoculation suggests low immunogenicity of CD46 in mice and validates the use of CD46-expressing mouse cell lines to study virus uptake and early virus transduction in mouse cells.

### Coinfection with MAV1 fails to complement genome replication or late protein expression of EnAd

**Fig. 6:**
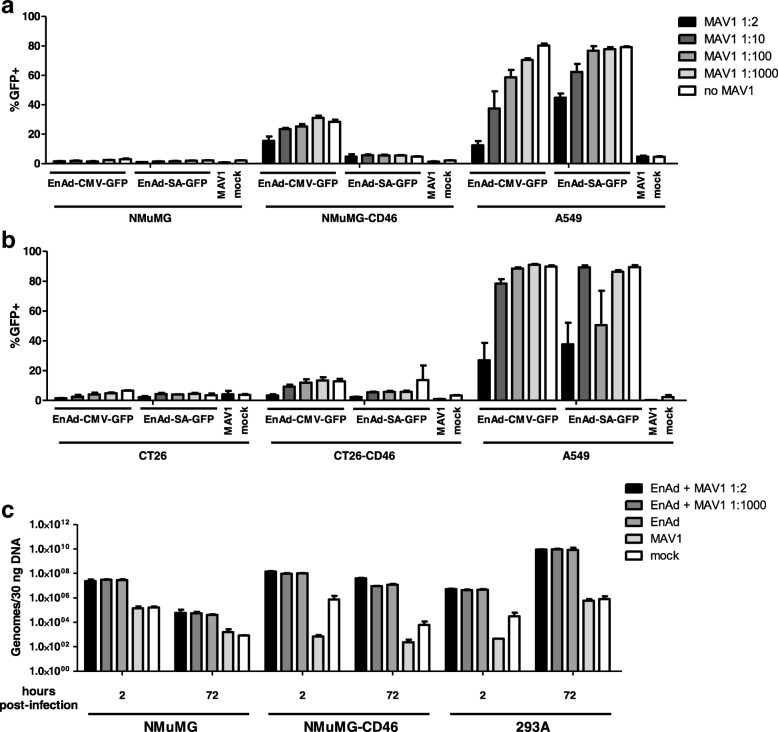
Coinfection with MAV1 fails to complement EnAd replication. **a** NMuMG, NMuMG-CD46, and A549 or (**b**) CT26, CT26-CD46, and A549 cells were seeded at 1 × 10^4^ cells/well in a 96-well plate before coinfection with crude MAV1-containing supernatant diluted at 1:2, 1:10, 1:100, or 1:1000 and 5000 virus particles/cell (VPC) of either EnAd-CMV-GFP or EnAd-SA-GFP, or mock-infected. Five days post-infection, GFP expression in cells was analysed by flow cytometry. **c** NMuMG, NMuMG-CD46, and 293A cells were coinfected with crude MAV1-containing supernatant diluted at 1:2 or 1:1000 and 5000 virus particles/cell of EnAd-CMV-GFP. At 2 and 72 h post-infection, cells were washed and harvested for genomic DNA extraction. EnAd genome copies in 30 ng of genomic DNA were quantified by qPCR using Taqman probes. Data represent biological triplicates, shown as mean ± SEM

Previous work by Young et al. suggested that coinfection with mouse adenovirus-1 could complement the replication defect of Ad5 in murine cells [18]. To determine whether MAV1 could also complement EnAd replication, murine and human cells were incubated with different dilutions of MAV1-containing supernatant and simultaneously with 5000 EnAd-CMV-GFP or EnAd-SA-GFP particles/cell or mock-infected. GFP expression was analysed by flow cytometry 5 days p.i. (Fig. 6a and b). Neither the expression of CMV promoter-driven GFP nor MLP-driven GFP expression was increased by the presence of MAV1. In contrast, the addition of large amounts of MAV1 (1:2 dilution of crude supernatant from infected CMT93 cells) actually lowered GFP expression in both cases, indicating either a level of cytotoxicity or possibly that MAV1 may be competing with EnAd for resources and niche establishment inside host cells. In addition EnAd genome replication remained unchanged in the presence or absence of MAV1 (Fig. 6c), indicating that MAV1 coinfection alone does not complement EnAd replication in murine cells.

### Coinfection with recombinant EnAd viruses containing ORFs from MAV1 does not enhance EnAd transgene expression controlled by the major late promoter

**Fig. 7:**
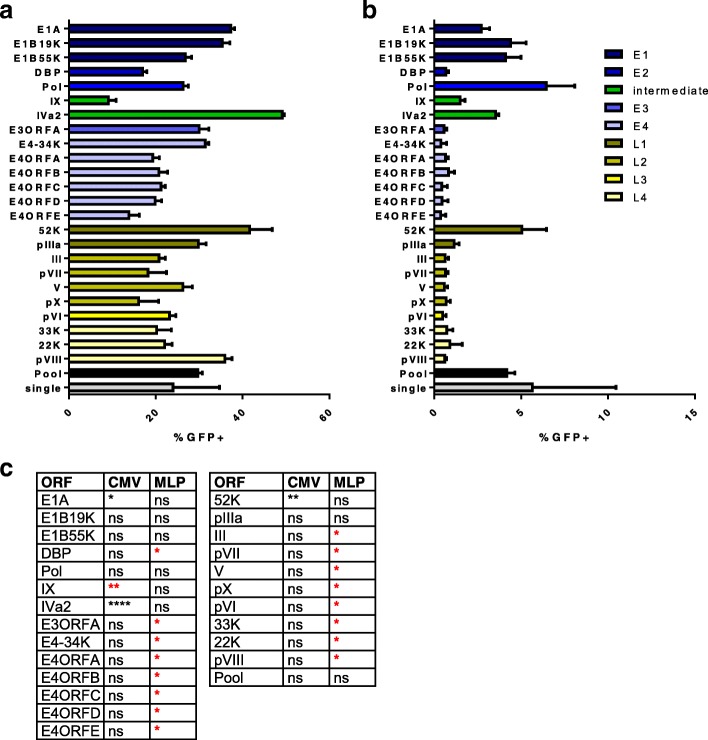
Several MAV1 genes improve CMV promoter-driven transgene expression in EnAd, but none increase virus replication. NMuMG-CD46 cells were seeded at 1 × 10^4^ cells/well in a 96-well plate before coinfection with 5000 virus particles/cell of either (**a**) EnAd-CMV-GFP or (**b**) EnAd-SA-GFP and recombinant EnAd clones expressing individual MAV1 genes under the control of the CMV promoter or a combination of all tested ORFs (‘pool’). Five days post-infection, GFP expression in the cells was quantified by flow cytometry. Data represent biological triplicates, shown as mean ± SEM. Blue bars represent early viral transcriptional units; green, intermediate; yellow, late. **c** Significance within each treatment was assessed using one-way ANOVA with Dunnett’s Post Hoc analysis compared to infection with either EnAd-CMV-GFP or EnAd-SA-GFP alone (‘single’). *, *p* < 0.05; **, *p* < 0.01; ****, *p* < 0.0001; ns, not significant. Black asterisks represent a significantly higher levels compared to single infections; red, significantly lower levels. ORF, open reading frame; CMV, cytomegalovirus immediate-early promoter; MLP, adenovirus major late promoter

As the observation that coinfection of murine cells with MAV1 was detrimental to transgene expression by EnAd, we tested whether individual MAV1 genes themselves could complement EnAd infection. By encoding each gene into EnAd, the potential problem of MAV1 competing for cellular resources can be circumvented. To test whether any of the MAV1 ORFs could complement late gene expression of EnAd in NMuMG-CD46 cells, 24 ORFs were expressed as transgenes under the control of a CMV promoter in EnAd (Additional files 1 and 2). These transgenic viruses were used to coinfect NMuMG-CD46 cells together with EnAd-CMV-GFP or EnAd-SA-GFP. Expression of GFP transgene expression was measured by quantifying the fraction of cells expressing green fluorescence using flow cytometry. NMuMG-CD46 cells were co-infected with 5000 VP/cell EnAd-CMV-GFP and each of the EnAd-CMV-MAV1-ORF-FLAG viruses (final concentration of all viruses, 5000 VP/cell). While most MAV1 ORFs did not have any effect on CMV-driven GFP expression, NMuMG-CD46 cells infected with EnAd encoding MAV1 E1A, IVa2, and 52 K had significantly higher levels of GFP than cells infected with EnAd-CMV-GFP alone (Fig. 7). Interestingly, NMuMG-CD46 cells infected with EnAd encoding MAV1 IX had significantly lower levels of GFP expression compared to cells infected with EnAd-CMV-GFP alone.

DNA-binding protein E3ORFA, pVI, and all E4, L2, L4 ORFs negatively affected MLP-driven GFP expression in NMuMG-CD46 cells. None of the ORFs tested positively influenced MLP-driven GFP expression. Therefore, we conclude that one or more of the MAV-1 proteins encoded in the pool of recombinant EnAd viruses can act to enhance simple EnAd-mediated GFP expression in murine cells, but none improves the virus life cycle sufficient to increase late gene expression.

## Discussion

Adenoviruses are one of the most widely used viral platforms for gene therapy and oncolytic virotherapy. Their genetic stability and transgene-encoding capabilities make them especially attractive for large-scale production and therapeutic applications. The increasing number of adenovirus-based oncolytic vaccines entering clinical trials has exposed a critical need for an immunocompetent murine model, ideally one that is broadly applicable to different adenovirus serotypes. Previous work trying to develop murine cells capable of supporting adenovirus replication have focussed almost exclusively on Ad5. For example, Hallden et al. showed that Ad5 could replicate efficiently in CMT64 cells in vitro and in vivo in C57BL/6 mice [7]. Similarly NMuMG murine breast epithelial cells have been reported to support Ad5 replication [6]. TC1-CD46 cells have also been used in an in vivo model for an Ad5/35 vector (Ad5 modified with an Ad35 fiber knob to target the virus to human CD46), though viral replication was also limited in this model [32].

Our study aimed to modify murine cells to support entry, transgene expression and replication of the chimeric group B adenovirus, EnAd. Initial studies showed that EnAd could infect and replicate a wide variety of human carcinoma cell lines but had no activity in CT26 murine carcinoma cells. Studies using EnAd-CMV-GFP showed that stable expression of human CD46 enabled virus to enter murine cells and achieve expression of reporter genes controlled by the CMV promoter, which is active immediately upon entry into the nucleus and does not depend on the adenovirus cycle of replication. However, studies using EnAd-SA-GFP showed that the presence of CD46 is not sufficient to allow the virus to progress through its life cycle sufficient to activate the MLP.

Though levels of E1A mRNA expression in NMuMG-CD46 cells were similar to levels in A549 cells, expression of E2B mRNA, part of the next transcriptional unit, is reduced in both NMuMG-CD46 and CT26-CD46 cells compared to A549. Unsurprisingly, this leads to knock-on effects of lower adenovirus genome replication, and decreased expression of fibre mRNA and structural proteins as well as decreased MLP-regulated expression of GFP. Co-infection with MAV-1 did not increase either CMV promoter-driven or MLP-driven GFP expression in EnAd, nor did it drive adenovirus genome replication. However, co-infection with recombinant EnAd encoding individual MAV-1 genes could sometimes enhance expression of CMV promoter-driven GFP but did not increase MLP-driven GFP. Some CD46-independent virus particle uptake was observed, possibly through heparan sulfate proteoglycans, though this uptake does not seem to lead to significant levels of CMV-driven GFP expression [33].

The surface expression levels of human CD46 we could achieve, even in clonally-selected cells, were lower in murine cells transduced with a lentiviral construct containing CMV-driven human CD46 gene compared to endogenous expression in A549 human cancer cells. This lower level of expression could be a consequence of several factors. First, lentivirus integration into an area of low transcriptional activity could be responsible for lower levels of expression, though puromycin resistance encoded on the same integron in the cells suggests that this is unlikely to be the explanation. It is also possible that human CD46 is not processed efficiently by the murine protein expression system, that the CD46 promoter becomes silenced through methylation (particularly if the CD46 is deleterious to the cell) or that the CD46 mRNA or protein has a shorter half-life in murine cells than in human. Our observation that there is substantial variation in the level of human CD46 that can be expressed in different cells fits with these latter possibilities.

Previous studies have attempted to alleviate the barriers to human adenovirus infection in murine cell lines. A study by Young et al. showed that MAV1 co-infection could complement Ad5 replication in MOVCAR7 cells [18]. However, our results show that MAV1 cannot complement replication of EnAd in any of the tested cell lines, suggesting that MAV1 may have differential transcomplementation abilities between group C and group B adenoviruses. This is supported by another study showing that NMuMG can support group C but not group B adenoviruses [6]. These studies support our conclusion that group B adenoviruses behave differently than group C adenoviruses within murine cells, potentially due to differences in cellular interaction partners, and that alleviation of factors allowing group C adenoviruses may not necessarily be valid for other adenovirus groups.

Adenovirus infection of human cells normally leads, after virus genome replication, to a switch from the classical cap-dependent 5′-3′ ribosome scanning to ribosome shunting, an alternative form of cap-dependent translation in which the ribosome skips large regions of mRNA to initiate translation at a downstream start codon. This occurs after virus early proteins are produced, using classical cap-dependent translation, and focuses the translational machinery on production of virus structural proteins via ribosome shunting. Young et al. observed that this switch is blocked for Ad5 in murine cells and could be partially alleviated by ectopic expression of human L4-100 K [18]. However, as co-infection of EnAd with MAV1 did not increase MLP-driven protein expression, a failure to activate ribosome shunting to enable late virus mRNA translation seems unlikely to entirely account for the lack of EnAd replication.

Our results suggest that the replication defect of group B adenoviruses stems from an overall inability to establish a cellular niche within infected cells that promotes strong expression of early genes and genome replication. Though there do not seem to be prohibitive differences in the ability of infected human and murine cells to transcribe E1A, the earliest gene in the virus replication cycle, levels of E2B mRNA (encoding the viral polymerase) are decreased in murine cells suggesting that the block to virus replication is mediated at an early stage. These lower mRNA levels explain the lower levels of virus genome observed in qPCR. We speculate that E1A from group B adenoviruses such as EnAd may be inactive in murine cells, allowing efficient expression of genes driven by the CMV and adenovirus E1A promoters, but not those driven by later promoters. A study of E1A protein sequences from different adenoviruses revealed a number of additional residues in human Ad2 and Ad5 E1A, including a 19-residue insertion, between conserved regions CR3 and CR4 [34]. Though no role has been identified for these extra residues, they could be involved in cellular interactions.

This project forms the basis for future studies on adapting murine cell lines to support human group B adenovirus replication. One possible approach for future development was exemplified by transcomplementation of Huh-7.5 cells with a human cDNA library that identified a single cDNA allowing pan-genotype replication of hepatitis C virus [35]. Human cDNA transcomplementation studies could also be applied here by transfecting murine cells with a human cDNA library to identify cDNAs that could render murine cells permissive to productive human adenovirus replication.

## Conclusions

Our results suggest that the absence of CD46 expression is only the first blockade to human group B adenovirus replication in murine cells. This study shows that human CD46 expression enables murine cells to be transduced with EnAd and that transgene expression can be driven by a replication-independent promoter such as CMV. Though much more work needs to be done to elucidate the inhibition mechanism, the inhibition occurs soon after entry of the virus into the nucleus and may reflect poor activity of E1A protein. Given the acute need for an immunocompetent murine model to evaluate the adaptive immune consequences of cancer immunotherapies encoded by oncolytic viruses, further studies are important to identify the inhibitory mechanisms preventing human group B adenovirus replication in murine hosts.

## Additional files

Additional file 1: Immunoblotting of FLAG-tagged MAV1 ORF transgenes encoded in EnAd. NMuMG-CD46 cells were infected with EnAd encoding different MAV1 ORFs as CMV-driven FLAG-tagged transgenes. **A.** Three days post-infection, cells were lysed and probed for the presence of the FLAG tag using a horseradish peroxidase-conjugated mouse anti-DYKDDDDK antibody. Red asterisks above bands denote proteins of approximately the predicted size for each transgene. Blots were probed with horseradish peroxidase-conjugated mouse anti-β-actin as a loading control. **B.** Predicted protein sizes for each ORF. (PPTX 1369 kb)

Additional file 2: RT-PCR of MAV1 ORF transgenes encoded in EnAd. CT26-CD46 and NMuMG-CD46 cells were infected with EnAd encoding different MAV1 ORFs as CMV-driven transgenes. Two days post-infection, total RNA was extracted from the cells and probed for the presence of mRNA encoding each respective transgene using primers binding to the 5-UTR and 3-UTR. Red boxes and asterisks denote the approximate predicted size of each ORF. **A**, **B**. RT-PCR using a melting temperature of 59 °C and an elongation time of 3 min (**A**) or 62 °C and 2 min (**B**). **C.** Predicted amplicon sizes for each ORF. (PPTX 2947 kb)

## Abbreviations

Ad5: Type 5 adenovirus
CAR: Coxsackie and adenovirus receptor
EnAd: Enadenotucirev
MAV: Murine adenovirus
MLP: Major late promoter
p.i.: Post-infection
