# Supplementary material for: Expression of human CD46 and trans-complementation by murine adenovirus 1 fails to allow productive infection by a group B oncolytic adenovirus in murine cancer cells
Source: J Immunother Cancer. 2018 Jun 13;6:55. doi: 10.1186/s40425-018-0350-x (PMC6000980; doi:10.1186/s40425-018-0350-x)
Supplement: Supplementary file 1 — Immunoblotting of FLAG-tagged MAV1 ORF transgenes encoded in EnAd. NMuMG-CD46 cells were infected with EnAd encoding different MAV1 ORFs as CMV-driven FLAG-tagged transgenes. A. Three days post-infection, cells were lysed and probed for the presence of the FLAG tag using a horseradish peroxidase-conjugated mouse anti-DYKDDDDK antibody. Red asterisks above bands denote proteins of approximately the predicted size for each transgene. Blots were probed with horseradish peroxidase-conjugated mouse anti-β-actin as a loading control. B. Predicted protein sizes for each ORF. (PPTX 1369 kb) [file 40425_2018_350_MOESM1_ESM.pptx]

## Slide 1
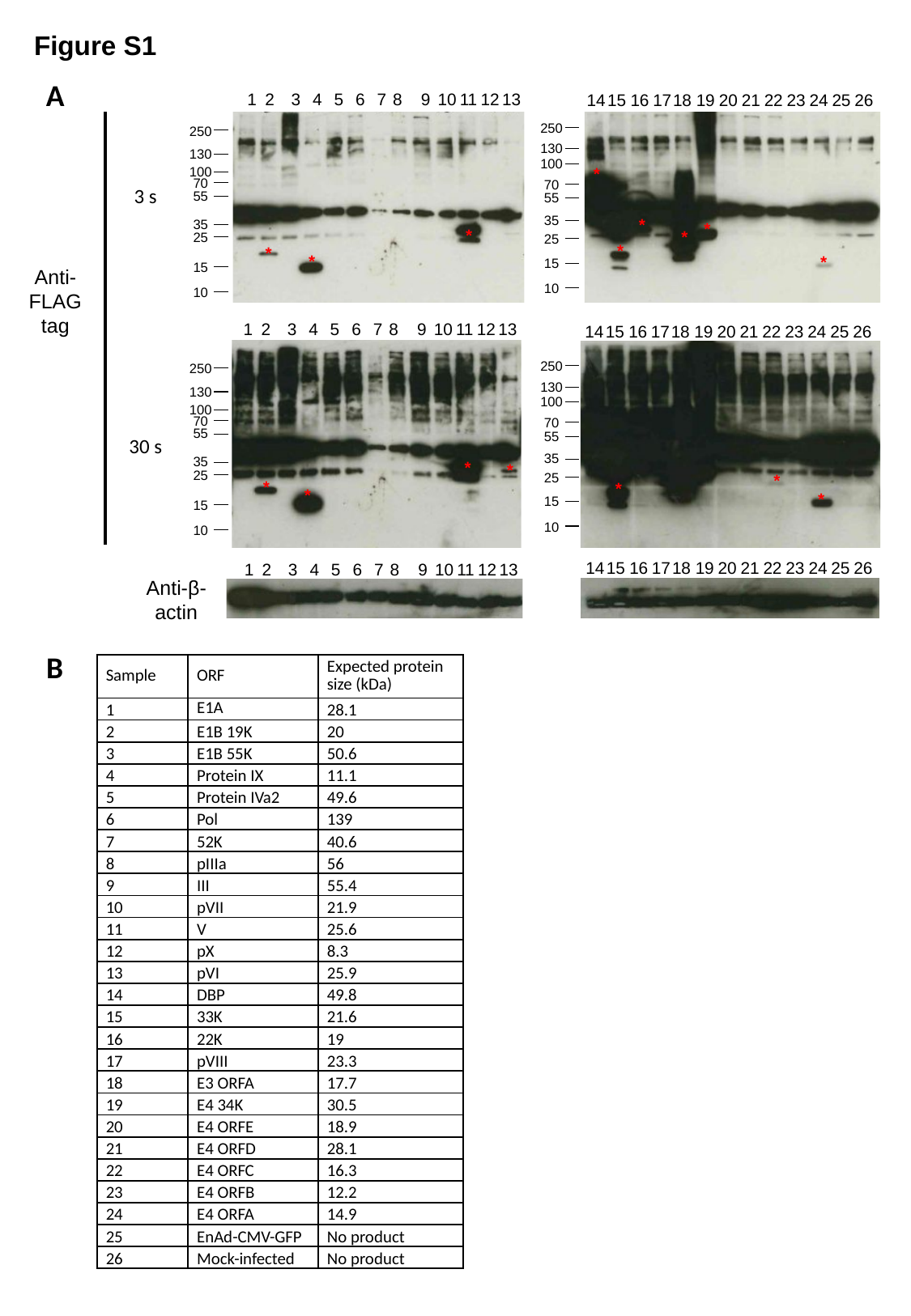

Figure S1
A
1
2
3
4
5
6
7
8
9
10
11
12
13
14
15
16
17
18
19
20
21
22
23
24
25
26
250
250
130
100
70
55
35
25
15
10
130
100
*
70
55
35
*
*
*
*
25
*
*
*
*
15
10
1
2
3
4
5
6
7
8
9
10
11
12
13
14
15
16
17
18
19
20
21
22
23
24
25
26
250
250
130
130
100
100
70
70
55
55
35
35
*
*
25
25
*
*
*
*
*
15
15
10
10
14
15
16
17
18
19
20
21
22
23
24
25
26
1
2
3
4
5
6
7
8
9
10
11
12
13
3 s
Anti-FLAG tag
30 s
Anti-β-actin
B
| Sample | ORF | Expected protein size (kDa) |
| --- | --- | --- |
| 1 | E1A | 28.1 |
| 2 | E1B 19K | 20 |
| 3 | E1B 55K | 50.6 |
| 4 | Protein IX | 11.1 |
| 5 | Protein IVa2 | 49.6 |
| 6 | Pol | 139 |
| 7 | 52K | 40.6 |
| 8 | pIIIa | 56 |
| 9 | III | 55.4 |
| 10 | pVII | 21.9 |
| 11 | V | 25.6 |
| 12 | pX | 8.3 |
| 13 | pVI | 25.9 |
| 14 | DBP | 49.8 |
| 15 | 33K | 21.6 |
| 16 | 22K | 19 |
| 17 | pVIII | 23.3 |
| 18 | E3 ORFA | 17.7 |
| 19 | E4 34K | 30.5 |
| 20 | E4 ORFE | 18.9 |
| 21 | E4 ORFD | 28.1 |
| 22 | E4 ORFC | 16.3 |
| 23 | E4 ORFB | 12.2 |
| 24 | E4 ORFA | 14.9 |
| 25 | EnAd-CMV-GFP | No product |
| 26 | Mock-infected | No product |
