# Supplementary material for: Expression of human CD46 and trans-complementation by murine adenovirus 1 fails to allow productive infection by a group B oncolytic adenovirus in murine cancer cells
Source: J Immunother Cancer. 2018 Jun 13;6:55. doi: 10.1186/s40425-018-0350-x (PMC6000980; doi:10.1186/s40425-018-0350-x)
Supplement: Supplementary file 2 — RT-PCR of MAV1 ORF transgenes encoded in EnAd. CT26-CD46 and NMuMG-CD46 cells were infected with EnAd encoding different MAV1 ORFs as CMV-driven transgenes. Two days post-infection, total RNA was extracted from the cells and probed for the presence of mRNA encoding each respective transgene using primers binding to the 5-UTR and 3-UTR. Red boxes and asterisks denote the approximate predicted size of each ORF. A, B. RT-PCR using a melting temperature of 59 °C and an elongation time of 3 min (A) or 62 °C and 2 min (B). C. Predicted amplicon sizes for each ORF. (PPTX 2947 kb) [file 40425_2018_350_MOESM2_ESM.pptx]

## Slide 1
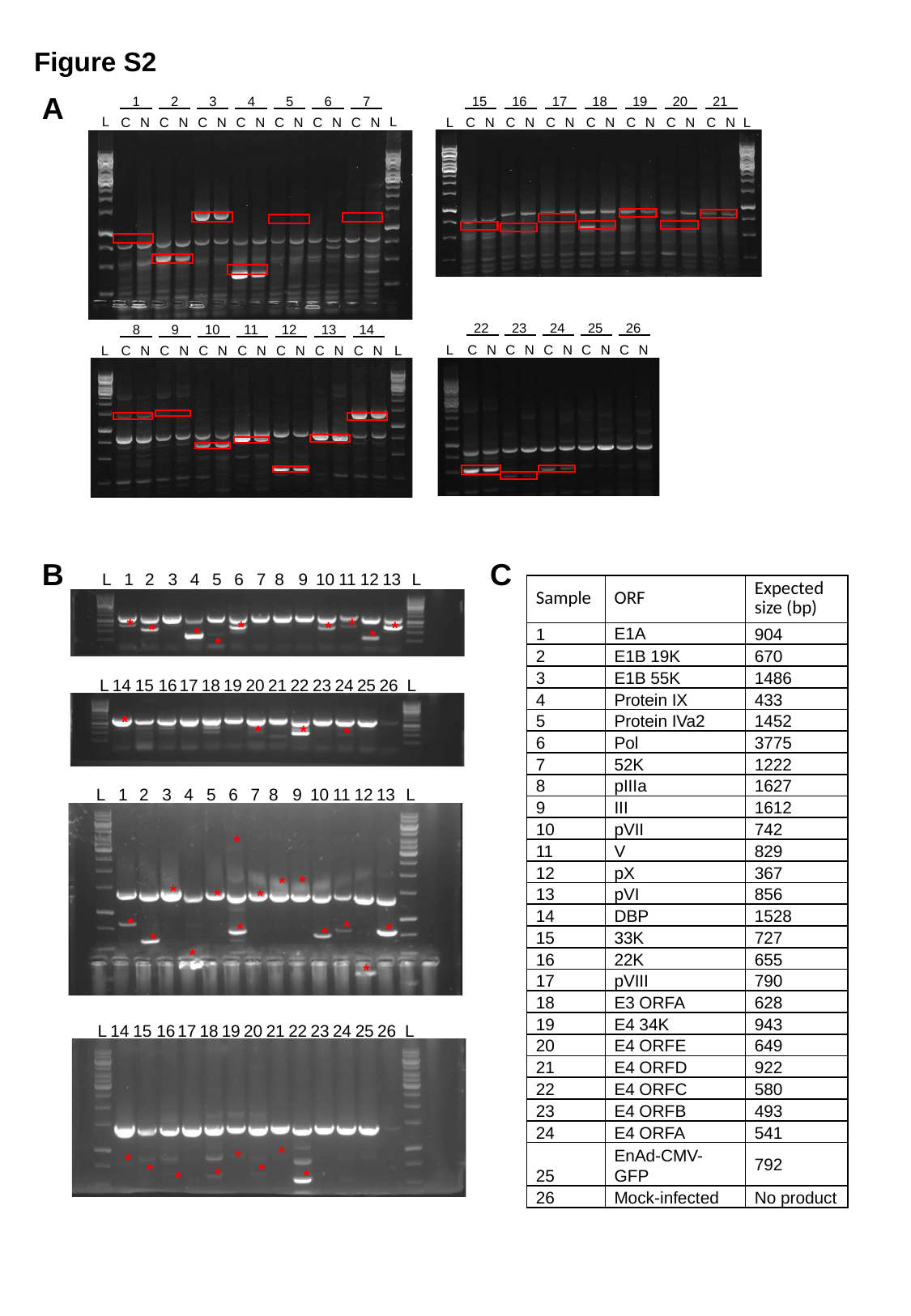

Figure S2
A
1
C
N
2
C
N
3
C
N
4
C
N
5
C
N
6
C
N
7
C
N
L
L
15
C
N
16
C
N
17
C
N
18
C
N
19
C
N
20
C
N
21
C
N
L
L
22
C
N
23
C
N
24
C
N
25
C
N
26
C
N
8
C
N
9
C
N
10
C
N
11
C
N
12
C
N
13
C
N
14
C
N
L
L
L
B
C
L
1
2
3
4
5
6
7
8
9
10
11
12
13
L
*
*
*
*
*
*
*
*
*
| Sample | ORF | Expected size (bp) |
| --- | --- | --- |
| 1 | E1A | 904 |
| 2 | E1B 19K | 670 |
| 3 | E1B 55K | 1486 |
| 4 | Protein IX | 433 |
| 5 | Protein IVa2 | 1452 |
| 6 | Pol | 3775 |
| 7 | 52K | 1222 |
| 8 | pIIIa | 1627 |
| 9 | III | 1612 |
| 10 | pVII | 742 |
| 11 | V | 829 |
| 12 | pX | 367 |
| 13 | pVI | 856 |
| 14 | DBP | 1528 |
| 15 | 33K | 727 |
| 16 | 22K | 655 |
| 17 | pVIII | 790 |
| 18 | E3 ORFA | 628 |
| 19 | E4 34K | 943 |
| 20 | E4 ORFE | 649 |
| 21 | E4 ORFD | 922 |
| 22 | E4 ORFC | 580 |
| 23 | E4 ORFB | 493 |
| 24 | E4 ORFA | 541 |
| 25 | EnAd-CMV-GFP | 792 |
| 26 | Mock-infected | No product |
L
14
15
16
17
18
19
20
21
22
23
24
25
26
L
*
*
*
*
L
1
2
3
4
5
6
7
8
9
10
11
12
13
L
*
*
*
*
*
*
*
*
*
*
*
*
*
*
L
14
15
16
17
18
19
20
21
22
23
24
25
26
L
*
*
*
*
*
*
*
*
